# Sex Distribution of Clinical Trial Participants: How Often Are Women Excluded?

**DOI:** 10.1007/s11606-025-09428-3

**Published:** 2025-02-19

**Authors:** Robert M. Kaplan, Sujin Song, Nikhil Majeti, Margaret C. Nikolov

**Affiliations:** Clinical Excellence Research Center, Stanford University School of Medicine, Center for Academic Medicine (CAM Building), Stanford, CA USA

Following the discovery that thalidomide taken during pregnancy resulted in serious limb deformities in newborns,^1^ there was a substantial decline in female participation in clinical trials. Generalizing the results from studies of men to women can have serious consequences. For example, men and women may metabolize hypnotic drugs differently. Using dosages based on men for prescribing zolpidem to women resulted in toxic overdoses.^2^ The 1993 NIH Revitalization Act required the agency to include women in each funded research project. The only exceptions were when inclusion could harm the health of women and when sex differences were not relevant to the research question.^3^ To investigate the inclusion of women in trials, we examined the sex distribution of participants in all clinical trials registered between 2000 and 2024.

## METHOD

We searched clinicaltrials.gov for all phase 3 interventional trials registered between the inception of the database in 2000 and January 1, 2024. Studies were coded for whether participants were women only, men only, or both sexes. The primary health condition or diagnosis for each study was also extracted. Each condition was classified as male- or female-specific if it explicitly related to male or female anatomy or if it predominantly affected one sex. The classification was independently replicated using CHAT-GPT, and discrepancies were adjudicated by the authors. Listings of the 27,218 trials and diagnoses are available on Harvard Dataverse (https://dataverse.harvard.edu/dataset.xhtml?persistentId=doi:10.7910/DVN/YPMZHK).

## RESULTS

The search identified 27,218 phase 3 interventional studies. Among these, 23,758 (87.3%) included both men and women. Women exclusively participated in 2559 studies (9.4%) while men were the exclusive focus of 885 studies (3.3%). Among the 653 studies that evaluated male-specific conditions, such as hemophilia, prostate disease, or erectile dysfunction, 91.1% included only men and 8.9% included both sexes. Among the studies of male-predominant conditions that included women, more than half (52%) considered hemophilia and female carriers. Others examined Fabry disease (22%) and Duchenne muscular dystrophy (5%). The remainder were descriptive studies that included partners of men with prostate disease or erectile dysfunction.

For the 1906 trials that evaluated female-specific conditions, such as breast cancer or uterine disease, 89.8% included only women and 10.2% included both sexes. Among the 194 studies that included both sexes, 92 (47%) focused on breast cancer, a condition that occasionally affects men. Other conditions in this category included pain, infertility, contraception, and HIV/sexually transmitted diseases. Only one trial concerning a female-specific condition included only male participants. That trial focused on breast cancer. Finally, 95.3% of the 24,659 studies of non–sex-specific conditions included both men and women (Table 1).

**Table 1:** Number and Percentage of Phase 3 Intervention Trials Registered in ClinicalTrials.gov Between 2000 and 2024

	Both sexes	Female only	Male only	Unspecified	Total
Non-specific	23,506 (95.3%)	848 (3.4%)	289 (1.2%)	16 (0.1%)	24,659 (100%)
Female-predominant	194 (10.2%)^a^	1711 (89.8%)	1 (0.1%)^b^	0 (0%)	1906 (100%)
Male-predominant	58 (8.9%)^c^	0 (0%)	595 (91.1%)	0 (0%)	653 (100%)
Total	23,758 (87.3%)	2559 (9.4%)	885 (3.3%)	16 (0.1%)	27,218 (100%)

Coding errors in ClinicalTrials.gov could not be ruled out. Rows: Number and percentage of trials for conditions that predominantly affect one or both sexes. Columns: Number and percentage of trials by sex of participants

^a^Inclusion of males in studies of female predominant diagnoses was most common for studies of breast conditions and may reflect male breast disease. In addition, the list includes diagnoses where male prevalence is low, but non-zero (i.e., lupus)

^b^Only 1 trial concerning a female-predominant condition exclusively included male participants. That trial focused on breast cancer. The protocol identified the condition as male breast cancer

^c^Many of the male predominant conditions were genetically linked where the phenotype is expressed primarily in males (i.e., hemophilia), but females can be carriers

## DISCUSSION

Clinical outcome studies that focus on a single sex should not be used to generalize to the opposite sex. Contrary to the putative suggestion that women are commonly excluded from trials,^4^ the great majority of phase 3 clinical trials included both men and women. Further, studies that focus exclusively on men almost always concentrate on organ systems or conditions that are peculiar to men or boys. Similarly, most studies that include only women or girls concentrate on organ systems or endocrine function unique to females. While the inclusion of women has generally improved,^5^ women remain underrepresented in cardiovascular trials.^6^ To better understand the appropriate use of heath care interventions, we need more studies that are powered to detect differences in responses of men and women.^7^

## LIMITATIONS

Our study has limitations. First, we only included studies registered in clinicaltrials.gov, a service that was not available before 2000. A significant portion of accumulated medical knowledge is based on older studies. Many of the drugs that remain commonly used were approved based on older trials that studied a disproportionate number of men. A second concern is that clinicaltrials.gov is primarily used to register US studies and may neglect key studies from other countries. Third, the use of clinicaltrials.gov has gradually increased over the last two decades. Studies from the first decade of the current millennium are less likely to have been registered. Finally, our study only coded whether the study included men or women, or both. For studies including both sexes, we were not able to determine the proportion of the total sample belonging to each sex. Future studies should evaluate whether studies have adequate statistical power to detect meaningful differences between the sexes.
